# Late cardiac sodium current can be assessed using automated patch-clamp

**DOI:** 10.12688/f1000research.5544.1

**Published:** 2014-10-16

**Authors:** Morgan Chevalier, Bogdan Amuzescu, Vaibhavkumar Gawali, Hannes Todt, Thomas Knott, Olaf Scheel, Hugues Abriel

**Affiliations:** 1 Department of Clinical Research, University of Bern, Bern, 3010, Switzerland; 2 Cytocentrics Bioscience GmbH, Rostock, 18059, Germany; 3 Medical University of Vienna, Wien, 1090, Austria; 4 Swiss National Centre of Competence in Research (NCCR) TransCure, University of Bern, Bern, 3010, Switzerland

## Abstract

The cardiac late Na ^+^ current is generated by a small fraction of voltage-dependent Na ^+^ channels that undergo a conformational change to a burst-gating mode, with repeated openings and closures during the action potential (AP) plateau. Its magnitude can be augmented by inactivation-defective mutations, myocardial ischemia, or prolonged exposure to chemical compounds leading to drug-induced (di)-long QT syndrome, and results in an increased susceptibility to cardiac arrhythmias. Using CytoPatch™ 2 automated patch-clamp equipment, we performed whole-cell recordings in HEK293 cells stably expressing human Nav1.5, and measured the late Na ^+^ component as average current over the last 100 ms of 300 ms depolarizing pulses to -10 mV from a holding potential of -100 mV, with a repetition frequency of 0.33 Hz. Averaged values in different steady-state experimental conditions were further corrected by the subtraction of current average during the application of tetrodotoxin (TTX) 30 μM. We show that ranolazine at 10 and 30 μM in 3 min applications reduced the late Na ^+^ current to 75.0 ± 2.7% (mean ± SEM, *n* = 17) and 58.4 ± 3.5% ( *n* = 18) of initial levels, respectively, while a 5 min application of veratridine 1 μM resulted in a reversible current increase to 269.1 ± 16.1% ( *n* = 28) of initial values. Using fluctuation analysis, we observed that ranolazine 30 μM decreased mean open probability *p* from 0.6 to 0.38 without modifying the number of active channels *n*, while veratridine 1 μM increased *n* 2.5-fold without changing *p*. In human iPSC-derived cardiomyocytes, veratridine 1 μM reversibly increased APD90 2.12 ± 0.41-fold (mean ± SEM, *n* = 6). This effect is attributable to inactivation removal in Nav1.5 channels, since significant inhibitory effects on hERG current were detected at higher concentrations in hERG-expressing HEK293 cells, with a 28.9 ± 6.0% inhibition (mean ± SD, *n* = 10) with 50 μM veratridine.   
**   **

## Introduction

The late cardiac Na ^+^ current can be recorded 10–100 milliseconds after membrane depolarization as a sustained inward current component ( *I _sus_*) in cardiomyocytes ^1^. This late current represents a fraction of voltage-dependent Na ^+^ channels that fail to inactivate after the initial opening. Instead, these channels change to a conformation with frequent late re-openings in a so-called burst mode ^2^. Among other mechanisms, calmodulin kinase II overexpression in chronic heart failure increases the rate of transition to the late bursting mode conformation ^2^. The phenomenon is also observed in Na _v_1.5 inactivation-deficient mutants, such as ΔKPQ ^3^ or 1795InsD ^4, 5^, leading to a specific form of congenital long QT syndrome, LQT-3 ^6– 8^.

Given its role in cardiac arrhythmogenesis ^9, 10^, pharmacological inhibition of the late Na ^+^ current component ( *I*_Na late_) is seen as an anti-arrhythmic strategy. Novel late sodium channel blockers, such as the partial fatty acid beta-oxidation inhibitor, ranolazine ^11– 15^, are used for both myocardial ischemia and to alleviate neuropathic pain and show a preferential affinity for the burst mode conformation of Na ^+^ channels ^16– 21^. Other compounds, such as veratridine, a steroid-derived alkaloid extracted from rhizomes of *Veratrum album* or seeds of *Schoenocaulon officinale* ^22, 23^, preferentially bind to activated Na ^+^ channels, impeding inactivation and leading to increased nerve excitability.

The objectives of this study were to (1) record the veratridine-dependent increase in the late Na ^+^ current using a HEK293 cell line stably transfected with human Na _v_1.5 ^24^, (2) measure the ranolazine-induced inhibition of basal and veratridine-activated *I*_Na late_, and (3) asses the effects of veratridine at the same concentrations on action potentials (APs) in hiPSC-cardiomyocyte preparations externally paced in current-clamp mode. In parallel we also tested the inhibitory effects of veratridine on hERG1 current in stably transfected HEK293 cells. All these experiments were performed using CytoPatch™2 automated patch-clamp equipment.

## Materials and methods

All experiments were performed using the CytoPatch™2, using standard dual-channel Cytocentrics chips with embedded quartz pipette tips 2 μM in diameter. For whole-cell late Na ^+^ current recordings, the voltage-clamp protocol consisted of repeated depolarizing pulses of -10 mV amplitude and 300 ms duration, from a holding potential of -100 mV, to allow a substantial removal from inactivation of Na _v_1.5 channels. The peak and late Na ^+^ current were plotted and monitored over the entire duration of experiment, and extracted from recorded data for further analysis. Pharmacological compounds were applied in a predefined sequence using the dispensing needle of automated equipment. All experiments were performed at room temperature (21–22°C).

For whole-cell hERG current recordings, cells were held at -70 mV. After a brief 100-ms prepulse to -50 mV to determine the current leak, a 2-s depolarizing voltage step to +40 mV was followed by a 2-s step to -50 mV to elicit hERG tail currents every 10 s. Peak tail current amplitude was corrected by the leak current and the corrected peak tail current was averaged over the last three pulses of the control phase ( *I*_ctrl_) and the application phase ( *I*_cpd_), respectively. From these averaged values the hERG tail current inhibition was calculated as follows:

          Inhibition = 1 – ( *I*_cpd_/ *I*_ctrl_)

iPSC-CM recordings were performed in current-clamp mode, using repeated sweeps consisting of 3 injected current pulses of 2000 pA amplitude, 0.5 ms duration, at 3-s intervals. During solution uptake the system was switched to voltage-clamp mode.

### Cell cultures and preparations

HEK293 cells stably expressing either the human Na _v_1.5 channel or the hERG K ^+^ channel were used as ready-to-use frozen Instant cells (product of Cytocentrics). Cells, kept in liquid nitrogen, were quickly thawed, centrifuged, resuspended in extracellular solution at a density of ~10 ^6^ cells/ml, and used for experiments within 3 hours. For other experiments, Nav1.5-expressing cells were cultured in 25 cm ^2^ flasks in DMEM supplemented with 10% fetal bovine serum and 100 μg/ml zeozin, and kept at 37°C, 8% CO _2_ in a humidified incubator. Adherent cell monolayers were detached with Versene (ethylenediaminotetraacetic acid – EDTA 0.02% in phosphate buffered saline - PBS), centrifuged for 2 min at 100 × g, and resuspended in hERG external solution.

hiPSC-derived iCell® Cardiomyocytes were kindly provided by Cellular Dynamics International (Madison, WI) as frozen samples, and cultured in monolayers in 12-well plates coated with 0.1% gelatin, for up to 41 days. The thawing/plating and maintenance media were provided by the cell supplier. For detachment, the monolayers were rinsed twice with calcium and magnesium-free PBS, then incubated for 2 min with trypsin 0.1% at 37°C. 1.5 ml of medium was added per well, the cells were suspended, centrifuged at 180 × g for 5 min, and resuspended in a 1:1 mixture of culture medium and hERG extracellular solution.

### Solutions and chemicals

For recordings in Na _v_1.5-transfected HEK293 cells the external solution had the following composition (in mM): NaCl 130, CsCl 5, CaCl _2_ 2, MgCl _2_ 1.2, HEPES 10, D-glucose 5, pH 7.4, osmolality 320 mOsm/kg. The internal solution contained (in mM): Cs-aspartate 70, CsCl 60, CaCl _2_ 1, MgCl _2_ 1, Na _2_ATP 5, EGTA 11, HEPES 10, pH 7.2 with CsOH, osmolality 290 mOsm/kg. For recordings in iPSC-derived cardiomyocytes and hERG-transfected HEK293 cells the extracellular solution had the following composition (in mM): 140 NaCl, 2.5 KCl, 2 MgCl _2_, 2 CaCl _2_, 10 HEPES, 10 Glucose, 15 Sucrose. The pH was adjusted to 7.4 with NaOH 1M and the osmolality to 320 (± 5) mOsmol/kg with sucrose 1M, and the storage temperature was 4°C. The intracellular solution contained (in mM): 100 K Gluconate, 20 KCl, 1 CaCl _2_, 1 MgCl _2_, 10 HEPES, 11 EGTA-KOH, 4 ATP-Mg ^2+^, 3 Phosphocreatine-Na _2_-H _2_O, 9 Sucrose. The pH was adjusted to 7.2 with KOH 1M, the osmolality to 295 (± 5) mOsmol/l, and then it was stored in 10-ml aliquots at -20°C, thawed and used within 4 hours. Veratridine (Sigma V5754) working solutions at 1, 5 and 50 μM were prepared from a 50 mM stock solution in ethanol. Ranolazine dihydrochloride (Sigma R6152) 10 and 30 μM was prepared from a 10 mM stock solution in DMSO. TTX (BN0518, Biotrend, Zurich, CH) was prepared from a 1 mM aqueous stock solution.

### Data storage and analysis

The software files generated during the recordings were stored on computer hard disks. Patch clamp data were analyzed using the CytoPatch ^TM^ software and exported to Microsoft Excel or pClamp10 (Axon Instruments, part of Molecular Devices, Sunnyvale, CA) for further analysis, using a proprietary conversion tool. APD90 analysis for current-clamp recordings in iPSC-derived cardiomyocytes was performed with self-written software routines. APD90 was computed as the duration between the point of maximal AP upstroke speed during phase 0 to recovery of 90% of the difference between peak upstroke potential and resting potential. In the case of veratridine application, if recovery was incomplete during the 3-s interstimulus interval, APD90 was computed over several pacing cycles. For statistical analysis, one-way ANOVA for independent samples with Dunnett’s post-hoc comparison, as well as Student’s *t* tests where appropriate, were applied, at a level of significance of *p* ≤ 0.05, using the GraphPad Prism software (La Jolla, CA).

## Results

### 1. Late Nav1.5 sodium current component

**Figure 1.**
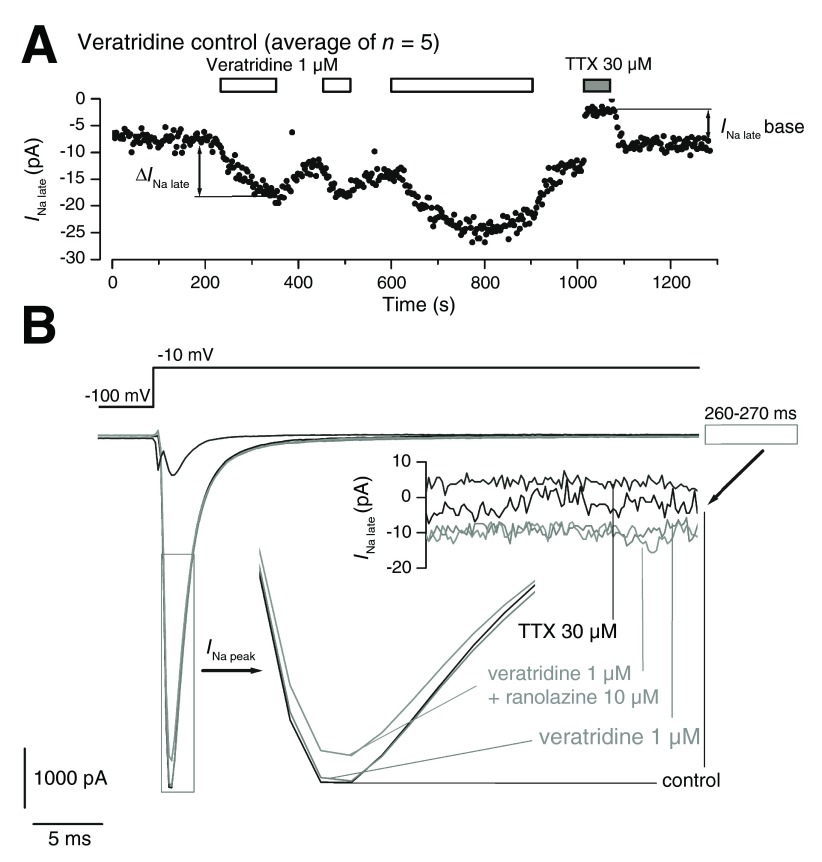
Activation by veratridine of the late Na ^+^ current in Nav1.5-expressing cells. **A**. Time course of *I*_Na late_ (average transmembrane current during the last 100 ms of a 300-ms depolarizing pulse to -10 mV from a holding potential of -100 mV) during a typical experiment with repeated applications of veratridine 1 μM and a final application of TTX 30 μM. **B**. Overlap of individual sweeps showing the peak and late component of *I*_Na_ during the application of veratridine 1 μM, the co-application with ranolazine 10 μM, and with TTX 30 μM. The peak and late *I*_Na_ component are shown separately in inserts using magnified time and voltage scales, respectively.

Using automated voltage-clamp protocols applied to human Nav1.5-expressing HEK293 cells in the above mentioned conditions, we routinely recorded, in high-quality seal conditions (both seal and membrane resistance > 1 GΩ), and with stability for at least 20 min, whole-cell Na ^+^ currents. These currents were elicited by membrane depolarization to -10 mV from a holding potential of -100 mV, required for the proper removal from inactivation of cardiac Na ^+^ channels. The late Na ^+^ current component ( *I*_Na late_) was automatically computed and plotted as time average over the last 100 ms of the 300-ms depolarizing pulse of each sweep. Figure 1A shows the time course of 5 averaged experiments including repeated applications of veratridine 1 μM with different durations (2 min, 1 min, 5 min), as well as a 1-min application of TTX 30 μM, resulting in the rapid complete block of late Na ^+^ current. The average current level during TTX application in each experiment was subsequently subtracted from all other averaged steady-state levels to obtain unbiased estimations of *I*_Na late_ in different experimental conditions. In general, reversibility was good and rapid upon TTX wash-out.

### 2. Late sodium current activation by veratridine and block by ranolazine in Nav1.5 cells

**Figure 2.**
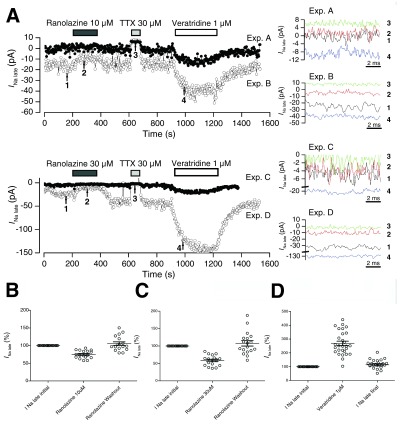
Effects of ranolazine, TTX and veratridine on the late Na ^+^ current in Nav1.5-expressing cells. **A**. Time plots of *I*_Na late_ in four typical experiments. Intervals of application of ranolazine 10 or 30 μM, TTX 30 μM, and veratridine 1 μM are indicated. Right panels: *I*_Na late_ traces from 260 to 270 ms at the time points marked with arrows and numbered in the left panels. **B** and **C**. Effects of ranolazine 10 and 30 μM, respectively, on relative levels of TTX-sensitive late Na ^+^ current. **D**. Effect of veratridine 1 μM. Mean values are indicated for each condition, and error bars represent SEM.

Under control conditions, a 5-min application of veratridine 1 μM induced an increase of the late Na ^+^ current by 269.1 ± 16.1% (mean ± SEM, *n* = 28) of initial values ( Table 1 and Figure 2). Figure 1B shows representative current traces of selected sweeps, as well as magnified inserts of the peak and late phase, within an individual experiment. The application of veratridine 1 μM increased the late current amplitude by more than two-fold relative to the TTX 30 μM reference trace, without any noticeable effect on peak current, while the co-application of ranolazine 10 μM reduced the peak current with apparently no effect on the late component. Systematic experiments with ranolazine applied alone demonstrated *I*_Na late_ reduction to 75.0 ± 2.7% (mean ± SEM, *n* = 17) of initial values at 10 μM, and 58.4 ± 3.5% (mean ± SEM, *n* = 18) at 30 μM ( Table 1 and Figure 2).

**Table 1.** Effects of ranolazine 10 and 30 μM and veratridine 1 μM on TTX-sensitive ^*^ late Na ^+^ current.

Experiment	Absolute values (mean ± SEM)			Relative values (mean ± SEM)				
Drug application ( *n* = number of cells tested)	***I*_Na late_ initial** (pA)	***I*_Na late_ drug** (pA)	***I*_Na late_ final** (pA)	***I*_Na late_ initial** (%)	***I*_Na late_ drug** (%)	***p*** value vs. initial ^§^	***I*_Na late_ final** (%)	***p*** value vs. initial ^§^
Ranolazine 10 μM 3 min ( *n* = 17)	-13.2 ± 2.6	-9.7 ± 1.8	-13.2 ± 2.3	100	75.0 ± 2.7	7.8E-8	105.6 ± 5.0	0.280
Ranolazine 30 μM 3 min ( *n* = 18)	-10.6 ± 2.2	-6.1 ± 1.4	-11.9 ± 2.8	100	58.4 ± 3.5	1.1E-9	107.7 ± 7.7	0.330
Veratridine 1 μM 5 min ( *n* = 28)	-12.4 ± 1.9	-36.3 ± 6.7	-15.8 ± 2.7	100	269.1 ± 16.1	4.7E-11	114.2 ± 8.2	0.095

^*^ *I*_Na late_ values were computed by subtraction of averaged amplitude in the presence of TTX 30 μM from averaged amplitudes in all other experimental conditions

^§^ using two-tailed Student’s *t* test for paired samples

**Figure 3.**
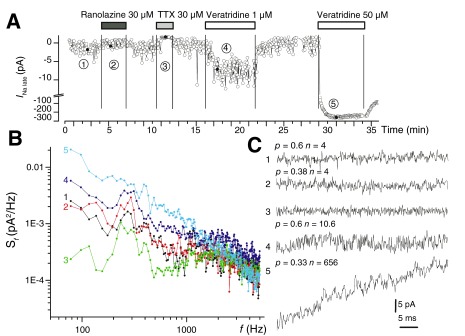
The late Na ^+^ current presents fluctuations due to the opening and closure of individual channels. **A**. Time course of a typical experiment in Nav1.5-expressing cells showing the effects of ranolazine 30 μM, TTX 30 μM, and veratridine 1 μM on *I*_Na late_. **B**. Averaged Fourier transforms of multiple traces recorded in different conditions in the experiment shown in **A**: 1. initial control; 2. ranolazine 30 μM; 3. TTX 30 μM; 4. veratridine 1 μM; 5. veratridine 50 μM. **C**. Fluctuation analysis of individual traces recorded during the five distinct periods shown in **A** allowed the distinction of individual channel gating events and the computation of the average open probability *p* and the average number of channels *n* for each condition.

Figure 3 shows the power density spectra of Fourier-transformed traces recorded under different conditions, as numbered in the upper time course of the experiments. Presumably, the opening and closure of Nav1.5 channels in the late gating mode conformation produces a Lorentzian component with corner frequency above 2 KHz. However, due to the reduced number of individual signal sources, the plateau spectral power density was small and difficult to distinguish. A better approach to fluctuation analysis is by the computation of single-channel parameters such as the mean open probability *p* and the average number of channels *n*, starting from the well-known estimations of current variance *σ* ^2^ and macroscopic current *I* ^25^:

*σ* ^2^ = *npi* ^2^ – *np* ^2^ *i* ^2^ = *np*(1 – *p*) *i* ^2^ and *I* = *npi*

We used a unitary Na ^+^ current amplitude *i* at -10 mV of 1.43 pA, based on the unitary current amplitude of 2 pA at -40 mV observed in single-channel recordings of Nav1.5 late currents in HEK293 cells ^26^ and a reversal potential of +65 mV. In doing so, we could evaluate the mean open probability *p* and further the average number of Na ^+^ channels *n* open in the late mode in selected traces, over the last 100 ms of activity during the 300-ms depolarizing pulses. It was observed that 30 μM ranolazine application resulted in a decrease of mean open probability from 0.6 to 0.38, without affecting the average number of channels contributing the late Na ^+^ current ( *n* = 4), in agreement with estimates obtained from averaged macroscopic currents. On the other hand, veratridine 1 μM increased the number of channels in late gating mode to *n* = 10, without influencing the mean open probability.

### 3. di-APD prolongation in iPSC-CM by application of veratridine

**Figure 4.**
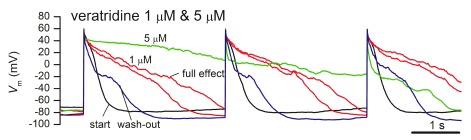
AP prolongation by veratridine in human iPSC-derived cardiomyocytes. Current-clamp AP recordings using a pacing protocol with injected current stimuli of 0.5-ms duration and 2 nA amplitude repeated at 3-s intervals. The overlap of traces in control conditions at the start of the experiment, with 1 μM veratridine (onset and full effect), with 5 μM veratridine, and during wash-out, shows partial recovery.

To get a better understanding of the role that the late Na ^+^ current may play in arrhythmogenesis and in the complex mechanisms of di-LQT syndrome, we performed a series of automated patch-clamp experiments on hiPSC-derived cardiomyocytes with current-clamp recordings of stimulus-triggered APs. Each recorded sweep contained responses elicited by three injected current stimuli (0.5 ms duration, 2 nA amplitude). As shown in Figure 4, veratridine 1 μM induced a marked prolongation of AP duration, reaching saturation in less than 3 min. Further application of 5 μM veratridine resulted in further APD90 prolongation, exceeding the interstimulus interval. There was a slow trend to reversibility during the wash-out phase. Quantitative results for veratridine 1 μM application are summarized in Table 2. Thus, in *n* = 6 experiments at room temperature the relative APD90 prolongation induced by veratridine was 2.12 ± 0.41 fold (mean ± SEM), reversible to 1.21 ± 0.50 fold at wash-out (mean ± SEM, *n* = 4).

**Table 2.** Changes in APD90 duration (in ms, mean ± SEM) induced by veratridine in hiPSC-derived cardiomyocytes.

Experimental condition	APD90 initial	APD90 during appl.	APD90 wash-out	Relative APD90 during appl.	Relative APD90 wash-out
Veratridine 1 μM	777.5 ± 179.1	1429.3 ± 289.8	688.0 ± 86.9	2.12 ± 0.41	1.21 ± 0.50
*p*		0.02482	0.65037		
*n*	6	6	4	6	4
Ethanol 0.1% control	893.8 ± 343.8	1013.0 ± 408.4	917.3 ± 318.3	1.08 ± 0.07	1.17 ± 0.17
*p*		0.16664	0.75986		
*n*	4	4	4	4	4

Relative values are computed taking initial values as reference

*p* values for Student’s *t* test for paired samples, two-tailed, for condition tested *vs.* initial values

*n* = number of cells included in analysis

### 4. Weak hERG inhibition by veratridine

**Figure 5.**
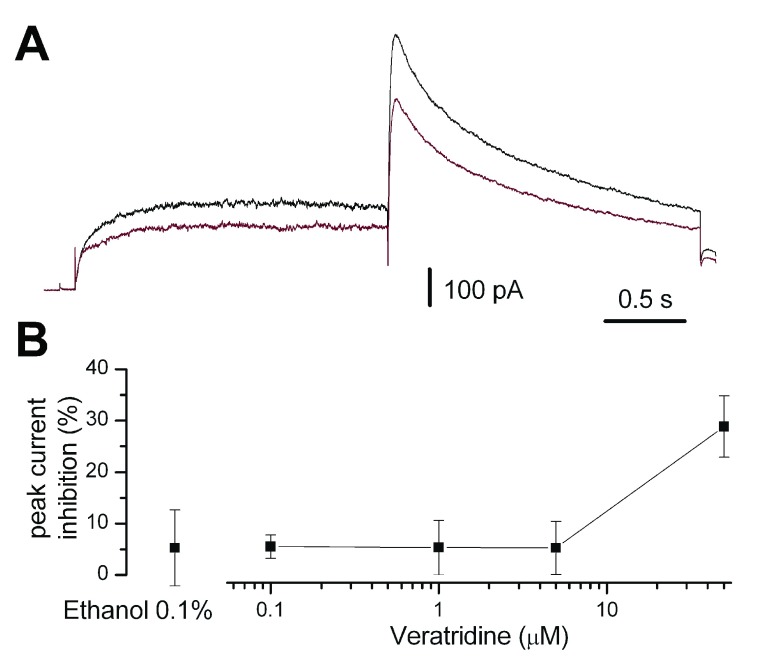
Effects of veratridine on hERG channels. **A**. Current traces in control conditions (upper) and during application of veratridine 50 μM (lower trace). **B**. Percentages of peak hERG current inhibition by different concentrations of veratridine and control ethanol 0.1% vehicle. Error bars represent SD. Number of experiments: control ( *n* = 6), veratridine 0.1 μM ( *n* = 10), 1 μM ( *n* = 5), 5 μM ( *n* = 7), 50 μM ( *n* = 10).

Last, we performed experiments using HEK293 cells stably expressing hERG1 to assess the effects of veratridine at different concentrations on this current component, in order to better understand the complex effects of this compound on the action potential of iPSC-derived cardiomyocytes. As shown in Figure 5, veratridine concentrations up to 5 μM did not produce levels of hERG inhibition significantly different than the diluting vehicle alone (ethanol 0.1%), while at 50 μM there was a 28.9 ± 6.0% (mean ± SD, *n* = 10) inhibition of peak hERG current, a statistically significant effect ( *p* < 0.0001, one-way ANOVA for independent samples with Dunnett’s multiple comparison test *vs*. control).

Experimental data showing the effect of veratridine, ranolazine and TTX on late Na ^+^ currents in cultured cells Patch-clamp data showing the effects on late Na ^+^ current after veratridine, ranolazine and TTX treatment in Nav1.5-expressing HEK293 cells, the effect of veratridine on hERG currents and on externally paced action potentials in iCell® Cardiomyocytes assessed with CytoPatch™2 automated patch-clamp equipment. For more details, please see the legend file in Dataset 1.

## Discussion

The main findings of the present study using the CytoPatch ^TM^2 instrument are the following: (1) inhibition of cardiac late Na ^+^ current to 75.0% and 58.4% of initial values by ranolazine 10 and 30 μM, respectively; (2) activation of the same current by veratridine 1 μM to 269.1% of initial values; and (3) prolongation of APD90 by veratridine 1 μM to 212% of initial values in human iPSC-derived cardiomyocytes. We have succeeded in recording and analysing the small (a few picoA) late Na ^+^ current component in a human cell line stably transfected with wild-type human SCN5A, using automated patch-clamp technology, with a voltage protocol different from that used in the first electrophysiology characterization of ΔKPQ LQT-3 mutant Na ^+^ channels ^3^. We observed that instrumental noise does not significantly affect the average current value over the last 100 ms of a 300-ms depolarizing pulse from -100 to -10 mV, therefore this value can be computed and plotted in real-time, representing a sensitive indicator for evidencing pharmacological effects in automated patch-clamp assays. Another critical parameter is the frequency of repetition of depolarizing stimuli, because it may reveal use dependency for compounds exerting state-dependent binding effects ^27^. Although in the present set of experiments we kept a fixed value of 0.33 Hz, inclusion of different pacing rates in future variants of the assay may offer supplementary information of pharmacological relevance. An important tool in the precise assessment of *I*_Na late_ levels is the application of TTX 30 μM, which results in an almost complete late Na ^+^ current inhibition, and offers a current reference used for the correction by subtraction of all other values in different experimental conditions. An excellent seal stability and access resistance, such as those offered by the Cytocentrics microfluidic chips ^28^, is another prerequisite for successful late Na ^+^ pharmacology experiments.

Due to the limited amplitude of late Na ^+^ current under basal conditions, we increased it using low concentrations of veratridine, a steroid-derived natural alkaloid known to bind to an intramembrane site of Na ^+^ channel pore subunits and, in doing so, stabilize it in the open conformation ^29, 30^, contributing to the *I*_Na late_. Although veratridine at 1 μM resulted in a consistent 2.5 – 3-fold increase in late Na ^+^ current, compared to initial levels ( Table 1 and Figure 1– Figure 3), we would not recommend this procedure for pharmacology assays because of possible interactions between veratridine and test compounds. Although we have not systematically explored this phenomenon, preliminary observations, as shown in Figure 1B, suggest a reduced effectiveness of ranolazine 10 μM when co-applied with veratridine, compared to application of ranolazine alone at the same concentration. The value of relative inhibition of the late Na ^+^ current by ranolazine 10 μM in the single compound application obtained in our study (75.0% of control levels) is larger than those obtained with the same concentration of ranolazine on late Na ^+^ current activated by veratridine 40 μM (89% and 81% for holding potentials of -110 mV and -90 mV, respectively) ^31^. An elegant method that may offer pharmacologically relevant information concerning the site and mechanism of action of a certain compound is fluctuation analysis. As shown in Figure 3, when applied to selected traces, this method allows the computation of the mean open probability *p* and the average number of open channels *n* contributing to current variance. From this analysis, we hypothesize that ranolazine acts as an open pore blocker, reducing the mean open probability at 30 μM to 63% of initial values, from 0.6 to 0.38, without lowering the number of active channels, while veratridine 1 μM increases the number of active channels in late gating mode 2.5-fold, without influencing their mean open probability.

In agreement with the significant activatory effects of veratridine 1 μM on cardiac Nav1.5 channels in late gating mode in stably transfected cell lines, we found an important prolongation of action potential duration when the drug was applied to human iPSC-derived cardiomyocytes in current-clamp configuration, using CytoPatch™2 automated patch-clamp equipment. The APD90 was increased over twofold by veratridine 1 μM, with a good reversibility upon wash-out ( Table 2), and an even higher prolongation with 5 μM ( Figure 4), although in this case we were unable to accurately compute APD90 values for externally paced APs, due to their overlap with the next stimulus. This effect is consistent with the findings of previous studies, showing APD prolongation in bronchial smooth muscle cells upon veratridine application ^32^, and the enhancement of AP burst generation by veratridine in rat trigeminal sensory neurons ^33^. Furthermore, we have demonstrated that hERG inhibition does not play any role in the observed APD prolongation by veratridine at 1 or 5 μM, since significant hERG inhibition occurred with higher veratridine concentrations ( Figure 5). A recent combined *in vivo* and *in vitro* study proved that the augmentation of the myocardial late Na ^+^ current during ventricular remodeling induced by pulmonary arterial hypertension in rats was accompanied by significant APD90 increases, and a beneficial effect of repeated ranolazine administration via late Na ^+^ current block was demonstrated with a subsequent alleviation of induced intracellular Ca ^2+^ overload ^34^.

In conclusion, the present study demonstrates that automated patch-clamp, as implemented by the CytoPatch™2 equipment using our proprietary CYTOCENTERING technology and quartz pipette tips embedded in silicon microfluidic chips ^28, 35^, which allow high-quality stable seals, lead to reliable late Na ^+^ current pharmacology recordings, with results at least comparable to those obtained in manual patch-clamp experiments. In addition, automation may prove advantageous given the small amplitude of late Na ^+^ current, which therefore requires a large number of experiments for the accurate assessment of pharmacological effects.

## Data availability

F1000Research: Dataset 1. Experimental data showing the effect of veratridine, ranolazine and TTX on late Na ^+^ currents in cultured cells, 10.5256/f1000research.5544.d36993 ^36^

## Abbreviations

HEK: human embryo kidney

CHO: Chinese hamster ovary

hERG: human ether-á-go-go related gene

SEM: standard error of the means

SD: standard deviation

DMEM: Dulbecco’s modified Eagle’s medium

DMSO: dimethylsulfoxide

HEPES: N-2-hydroxyethylpiperazine-N´-2-ethansulfonic acid

EGTA: ethylene glycol-bis(ß-aminoethyl ether)-N,N,N,N´-tetraacetic acid

ATP: adenosine triphosphate

TTX: tetrodotoxin

IC: intracellular solution (pipette-filling solution)

EC: extracellular solution

PBS: phosphate-buffered saline

iPSC-CM: induced pluripotent stem cell-derived cardiomyocytes

APD: action potential duration
