# Supplementary material for: Late cardiac sodium current can be assessed using automated patch-clamp
Source: F1000Res. 2014 Oct 16;3:245. [Version 1] doi: 10.12688/f1000research.5544.1 (PMC4215750; doi:10.12688/f1000research.5544.1)
Supplement: Experimental data showing the effect of veratridine, ranolazine and TTX on late Na + currents in cultured cells — Patch-clamp data showing the effects on late Na + current after veratridine, ranolazine and TTX treatment in Nav1.5-expressing HEK293 cells, the effect of veratridine on hERG currents and on externally paced action potentials in iCell® Cardiomyocytes assessed with CytoPatch™2 automated patch-clamp equipment. For more details, please see the legend file in Dataset 1. [file f1000research-3-5923-s0000.tgz › 731516aa-b41e-4465-9645-d2076c0a641a_Dataset1.zip!/legend.docx]

**Dataset.Fig1A** - I Na late in 5 Nav1.5-expressing HEK293 cells, with 3 applications of veratridine 1 µM, lasting 2, 1, and 5 min, respectively.

**Dataset.Fig1B** - I Na peak and I Na late recorded in a HEK293 cell expressing Nav1.5 in control conditions (column B), with veratridine 1 µM (column B), veratridine 1 µM and ranolazine 10 µM (column C), and TTX 30 µM (column D).

**Dataset.Fig2A** - I Na late in 4 Nav1.5-expressing HEK293 cells, with consecutive application of ranolazine 10 µM (columns B,C) or 30 µM (columns D,E) for 3 min, TTX 30 µM for 1 min, and veratridine 1 µM for 5 min.

**Dataset.Fig2BCD** - Percentage of I Na late activation/inhibition by ranolazine 10 and 30 µM and veratridine 1 µM in Nav1.5-expressing HEK293 cells.

**Dataset.Fig3A** - Time course of peak and late Na current in a HEK293 cell expressing Nav1.5, used for fluctuation analysis.

**Dataset.Fig3B** - Fourier-transformed power density spectra of late Na current traces (last 100 ms of 300-ms depolarizing pulses), averaged over multiple traces, in control conditions (columns B,C), ranolazine 30 µM (columns D,E), TTX 30 µM (columns F,G), veratridine 1 µM (columns H,I), and veratridine 50 µM (columns J,K) for Na currents recorded in a Nav1.5-expressing HEK293 cell.

**Dataset.Fig3C** - I Na late recorded in several sweeps, in control conditions (column B), with ranolazine 30 µM (column C), TTX 30 µM (column D), veratridine 1 µM (column E), and veratridine 50 µM (column F), used for fluctuation analysis, in a Nav1.5-expressing HEK293 cell.

**Dataset.Fig4** - Transmembrane potential recordings in an iCell® Cardiomyocyte paced with 2 nA 0.5 ms stimuli at 3-s intervals in control conditions (column B), veratridine 1 µM (columns C,D), veratridine 5 µM (column E), and wash-out (column F).

Dataset.Fig5A - Time course of transmembrane current in a hERG1-expressing HEK293 cell in control conditions (column B) and during application of veratridine 50 µM (column C).

**Dataset.Fig5B** - Relative inhibition of peak current in hERG-expressing HEK293 cells with different concentrations of veratridine (mea n± SD; n=number of cells tested).
