# Supplementary material for: Late cardiac sodium current can be assessed using automated patch-clamp
Source: F1000Res. 2014 Oct 16;3:245. [Version 1] doi: 10.12688/f1000research.5544.1 (PMC4215750; doi:10.12688/f1000research.5544.1)
Supplement: Peer review history

# Peer review: Referee response for version 1

**DOI:** 10.5256/f1000research.5923.r6444

**Published:** 2014-10-29

**Authors:** Céline Fiset

**Affiliations:** 1 Research Center, Montreal Heart Institute and Faculty of Pharmacy, Université de Montréal, Montréal, Canada

In this study, Chevalier *et al.* had succeeded to record and analyse the late Na ^+^ current (I _NaL_) in HEK293 cells stably transfected with wild-type human SCN5A, using automated patch-clamp technology. They were able to accurately assess the effects of pharmacological agents on I _NaL_. They showed that the cardiac I _NaL_ can be inhibited by ranolazine and activated by veratridine. Using fluctuation analysis, they hypothesized that ranolazine would act as an open pore blocker, reducing the open probability without affecting the number of active channels, while veratridine would increase the number of active channels without influencing their open probability. In addition, they also provided additional functional data by performing a series of automated patch-clamp experiments showing that application of veratridine concentrations that increased I _NaL_ but had no effects on HERG current prolonged the action potential duration of human iPSC-derived cardiomyocytes.

This is an interesting and well-designed study that reports high quality data. Considering the role of late sodium current in the action potential duration, mainly under pathological conditions, and the difficulty to accurately record this small ionic current, the subject of this study is highly relevant. The demonstration of the suitability of the automated patch clamp technology to accurately measure the late sodium current and to assess the effects of pharmacological agents on this current is helpful. In addition, the use of the hiPSC –derived cardiomyoyctes with this approach is also interesting. I do not have any major issues to address. However, the paper could be improved by attention to the following points.

 

1.  All experiments were performed at room temperature. Considering the limited amplitude of the late Na ^+^ current under basal conditions, it could have been useful to perform these experiments at a more physiological temperature to help increase the amplitude of the current.
2. In Figure 1B, it would have been interesting to also present the recordings with ranolaxine alone.
3. The *action potential *was elicited by the *injection *of 2 nA of *current*. This is a rather large current and is probably not just-suprathreshold depolarizing *current*. Do the hiPSC-derived cardiomyocytes usually require such a high current injection to elicit action potential?
4. For the HERG recordings, is there any specific reason why the external concentration of KCl was only 2.5 mM?
5. Data reported in Table 1 could have been normalized to the cell capacitance to report the current density instead of the current amplitude.
6. For some statistical analysis, an ANOVA would have been more appropriate that a Student’s *t *tests as several groups were examined (eg., Tables 1 and 2).
7. There are few typographic errors in the Abstract: the units for the drugs ranolaxine, veratridine and TTX are all in millimolar concentrations but should be in the micromolar concentrations.

Overall, the study is interesting and describes a methodological approach that could help study the late sodium current. The experiments were well designed and presented and the subject of this manuscript is of importance to the field.

I have read this submission. I believe that I have an appropriate level of expertise to confirm that it is of an acceptable scientific standard.

# Peer review: Referee response for version 1

**DOI:** 10.5256/f1000research.5923.r6442

**Published:** 2014-10-28

**Authors:** Eva Delpon, Ricardo Caballero

**Affiliations:** 1 Facultad de Medicina, Universidad Complutense de Madrid, Madrid, Spain

The paper by Chevalier *et al*. analyzed whether late sodium current (I _NaL_) can be assessed using an automated patch-clamp device. To this end, the I _NaL_ effects of ranolazine (a well known I _NaL_ inhibitor) and veratridine (an I _NaL_ activator) were described. The authors tested the CytoPatch automated patch-clamp equipment and performed whole-cell recordings in HEK293 cells stably transfected with human Nav1.5. Furthermore, they also tested the electrophysiological properties of human induced pluripotent stem cell-derived cardiomyocytes (hiPS) provided by Cellular Dynamics International. The title and abstract are appropriate for the content of the text. Furthermore, the article is well constructed, the experiments were well conducted, and analysis was well performed.

I _NaL_ is a small current component generated by a fraction of Nav1.5 channels that instead to entering in the inactivated state, rapidly reopened in a burst mode. I _NaL_ critically determines action potential duration (APD), in such a way that both acquired (myocardial ischemia and heart failure among others) or inherited (long QT type 3) diseases that augmented the I _NaL_ magnitude also increase the susceptibility to cardiac arrhythmias. Therefore, I _NaL_ has been recognized as an important target for the development of drugs with either antiischemic or antiarrhythmic effects. Unfortunately, accurate measurement of I _NaL_ is a time consuming and technical challenge because of its extra-small density. The automated patch clamp device tested by Chevalier **et al.** resolves this problem and allows to fast and reliable I _NaL_ measurements.

The results here presented merit some comments and arise some unresolved questions. First, in some experiments (such is the case in experiments B and D in Figure 2) current recordings obtained before the ranolazine perfusion seem to be quite unstable. Indeed, the amplitude progressively increased to a maximum value that was considered as the control value (highlighted with arrows). Can this problem be overcome? Is this a consequence of a slow intracellular dialysis? Is it a consequence of a time-dependent shift of the voltage dependence of activation/inactivation? Second, as shown in Figure 2, intensity of drug effects seems to be quite variable. In fact, experiments A, B, C, and D in Figure 2 and panel 2D, demonstrated that veratridine augmentation ranged from 0-400%. Even assuming the normal biological variability, we wonder as to whether this broad range of effect intensities can be justified by changes in the perfusion system. Has been the automated dispensing system tested? If not, we suggest testing the effects of several K ^+^ concentrations on inward rectifier currents generated by Kir2.1 channels (I _Kir2.1_).

The authors demonstrated that the recording quality was so high that the automated device allows to the differentiation between noise and current, even when measuring currents of less than 5 pA of amplitude. In order to make more precise mechanistic assumptions, the authors performed an elegant estimation of current variance (σ ^2^) and macroscopic current (I) following the procedure described more than 30 years ago by Van Driessche and Lindemann ^1^. By means of this method, Chevalier et al. concluded that ranolazine acts as an open pore blocker reducing the open channel probability, while veratridine increases the number of channels in the burst mode. We respectfully would like to stress that these considerations must be put in context from a pharmacological point of view. We do not doubt that ranolazine acts as an open channel blocker, what it seems clear however, is that its onset block kinetics has to be “ultra” slow, otherwise ranolazine would decrease peak I _Na_ even at low frequencies of stimulation. This comment points towards the fact that for a precise mechanistic study of ionic current modifying drugs it is mandatory to analyze drug effects with much more complicated pulse protocols. Questions thus are: does this automated equipment allow to the analysis of the frequency-, time-, and voltage-dependent effects of drugs? Can versatile and complicated pulse protocols be applied? Does it allow to a good voltage control even when generated currents are big and fast? If this is not possible, and by means of its extraordinary discrimination between current and noise, this automated patch-clamp equipment will only be helpful for rapid I _NaL_-modifying drug screening. Obviously it will also be perfect to test HERG blocking drug effects as demanded by the regulatory authorities.

Finally, as cardiac electrophysiologists, we would like to stress that it seems that our dream of testing drug effects on human ventricular myocytes seems to come true. Indeed, human atrial myocytes are technically, ethically and logistically difficult to get, but human ventricular are almost impossible to be obtained unless from the explanted hearts from patients at the end stage of cardiac diseases. Here the authors demonstrated that ventricular myocytes derived from hiPS generate beautiful action potentials that can be recorded with this automated equipment. The traces shown suggested that there was not alternation in the action potential duration. Is this a consistent finding? How long do last these stable recordings? The only comment is that resting membrane potential seems to be somewhat variable. Can this be resolved? Is it an unexpected veratridine effect? Standardization of maturation methods of ventricular myocytes derived from hiPS will be a big achievement for cardiac cellular electrophysiology which was obliged for years to the imprecise extrapolation of data obtained from a combination of several species none of which was representative of human electrophysiology. The big deal will be the maturation of human atrial myocytes derived from hiPS that fulfil the known characteristics of human atrial cells.

**Minor points:**

We suggest suppressing the initial sentence of section 3. We surmise that results obtained from the experiments described in this section cannot serve to understand the role of I _NaL _in arrhythmogenesis.

We have read this submission. We believe that we have an appropriate level of expertise to confirm that it is of an acceptable scientific standard.

## References

[1] Van DriesscheWLindemannB: Concentration dependence of currents through single sodium-selective pores in frog skin.*Nature*.1979;282(5738) : 245519-52010.1038/282519a0315521
